# Characterization of spatio-temporal epidural event-related potentials for mouse models of psychiatric disorders

**DOI:** 10.1038/srep14964

**Published:** 2015-10-13

**Authors:** Xin Wang, António Pinto-Duarte, M. Margarita Behrens, Xianjin Zhou, Terrence J. Sejnowski

**Affiliations:** 1 Howard Hughes Medical Institute and the Salk Institute for Biological Studies, La Jolla, CA 92037, USA; 2 Department of Psychiatry, University of California at San Diego, La Jolla, CA 92093, USA; 3 Division of Biological Sciences, University of California at San Diego, La Jolla, CA 92093, USA

## Abstract

Distinctive features in sensory event-related potentials (ERPs) are endophenotypic biomarkers of psychiatric disorders, widely studied using electroencephalographic (EEG) methods in humans and model animals. Despite the popularity and unique significance of the mouse as a model species in basic research, existing EEG methods applicable to mice are far less powerful than those available for humans and large animals. We developed a new method for multi-channel epidural ERP characterization in behaving mice with high precision, reliability and convenience and report an application to time-domain ERP feature characterization of the *Sp4* hypomorphic mouse model for schizophrenia. Compared to previous methods, our spatio-temporal ERP measurement robustly improved the resolving power of key signatures characteristic of the disease model. The high performance and low cost of this technique makes it suitable for high-throughput behavioral and pharmacological studies.

Impairments in sensory processing and perception are well-established endophenotypes of psychiatric disorders^1^,^2^, and could be empirically characterized by distinctive features of event-related potentials (ERPs) in electroencephalographic (EEG) signals^2^,^3^,^4^,^5^,^6^,^7^. Specifically, auditory ERPs are among the most widely studied markers, thanks to decades of accumulated clinical data^8^. ERPs can be conveniently measured in model animals as well as in humans^3^,^9^,^10^,^11^,^12^.

Many reverse-translated mouse models of psychiatric disorders have been recently introduced, exhibiting a broad spectrum of genetic and environmental etiologies^13^,^14^,^15^,^16^. Although mouse models have been used to better understand the neural circuit mechanisms underlying ERP anomalies associated with psychiatric disorders, it has been difficult to infer homology between the human and the murine ERP features^3^. This difficulty is, in a substantial part, methodological, due to the lack of powerful EEG methods for ERP measurements in mice. Though ERPs have been extensively studied in mice^17^,^18^,^19^,^20^,^21^,^22^,^23^, commonly used mouse ERP methods in basic research laboratories typically have poor spatial resolution, substantial inter-subject variability, low resolving power of relevant brain states, procedural complexity and high cost in up-scaling for large studies, in comparison with standard techniques for human subjects. Richer and higher quality EEG data generated at a larger scale from behaving mice would allow a better understanding of the translational relationship of homologous EEG phenotypes between humans and mice, and those between different mouse models.

We describe a new method of epidural EEG recording from multiple cortical surface loci in behaving mice, integrating a minimalistic electrode implant design and an optimized surgical procedure. Our technique has high stereotaxic precision, low variability across subjects, stability over time, procedural convenience and low cost, compared with extant methods. Thus, our methods have the potential to strengthen experimental studies of reverse-translated mouse models of psychiatric disorders.

## Results

**Figure 1:**
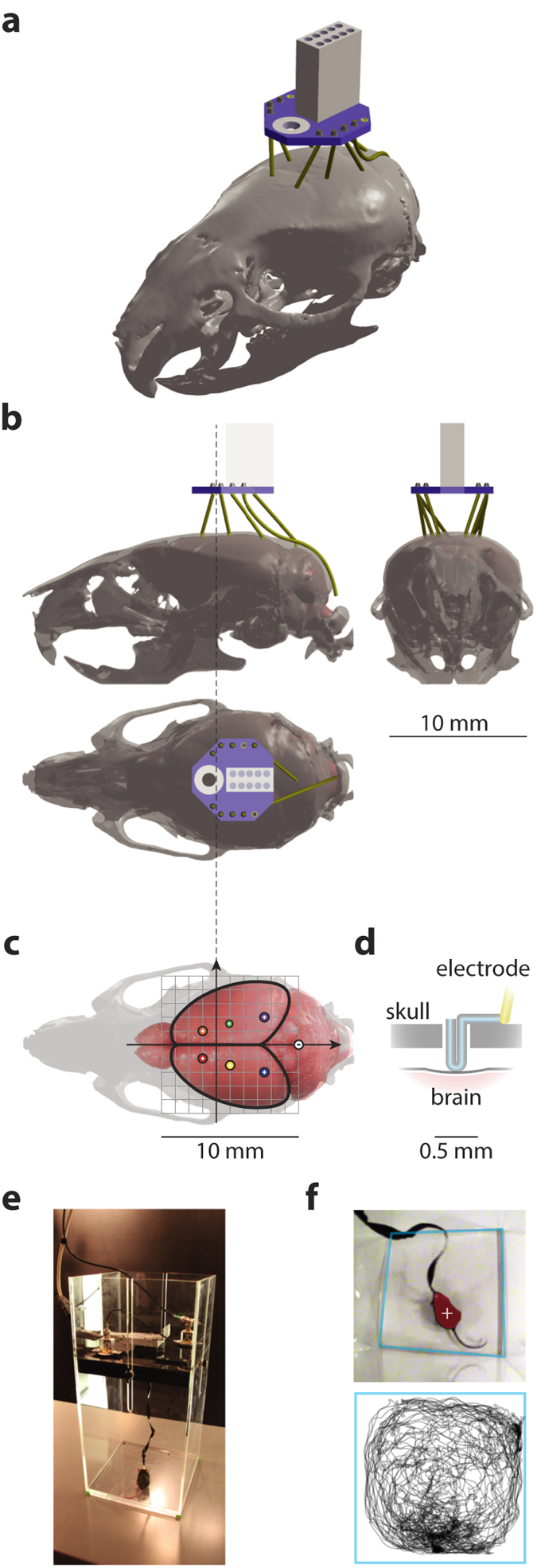
Physical setup of the recording apparatus. (**a**) A 3D projective rendering of the implant device on top of the skull of an adult C57Bl/6J mouse with epidural electrodes resting in craniotomies of the exact stereotaxic coordinates. For clarity, jewelry screws and dental acrylic for mechanical fixation were omitted from the illustration. The implanted electrodes (yellow) were interfaced through a board (Neuralynx EIB-8, blue) with an Omnetics nano connector (white). The implant contained in total 7 epidural EEG electrodes and 1 subcutaneous EMG electrode. 3D model assembled from components obtained from the Henderson 3D surgical atlas 24 and the EIB-8 model from Neuralynx (see Methods for procedural details of the physical assembly). (**b**) Three-view diagrams of the assembly of the skull (transparent gray) and brain (pink solid) with the 3D model of the implant device. (**c**) A top-view of the positioning of epidural electrodes (colored circles, recording electrodes labeled with a plus sign and the reference electrode a minus sign) on the surface of the brain (pink). The scale of this diagram is exactly the same as in (**b**); a millimeter grid (gray) is overlaid on top of the brain and solid arrows represent the major anatomical axes; the extending dashed line mark the antero-posterior position of bregma. (**d**) A diagram of a vertical section of a craniotomy showing how an epidural electrode rests on the cranium with its tip contacting the dural surface. Insulation (yellow) of the electrode wire was stripped at the tip. (**e**) A 25 by 25 cm^2^ recording chamber designed for the experiments, picture showing a C57Bl/6J mouse being tested. A wired tether, interfaced through a pre-amplifier and a slip-ring commutator at its two ends, carried recording signals; two passive speakers and a camera were mounted on the ceiling of the chamber to deliver sensory stimuli and to monitor animal behavior. (**f**) A frame captured by the monitoring camera (top), showing tracking of the center-of-mass (white cross) of the detected animal (red shade) in an automated way. Boundaries of the recording chamber (light blue) were detected offline so that the real-world coordinates of the animal’s positions could be reconstructed (bottom, showing the trajectory of the animal during an hour).

We designed a minimalistic epidural EEG electrode implant aided by the Henderson 3D surgical atlas of adult mice^24^ (Fig. 1a–c), developed an optimized surgical procedure and built an experimental setup for ERP characterization in behaving animals (Fig. 1e,f). For technical details see Methods.

**Figure 2:**
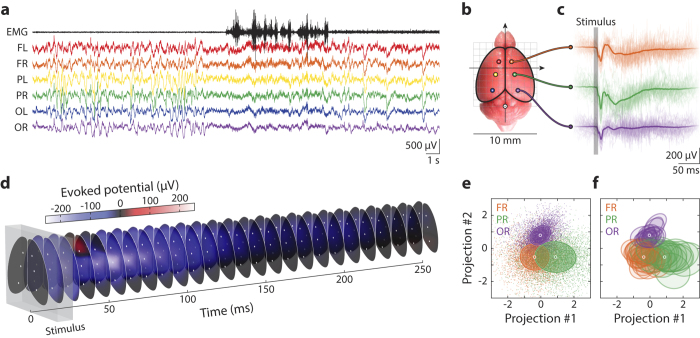
Recordings of spatio-temporal auditory ERPs from behaving mice at high accuracy, precision and reliability. (**a**) A 30-second clip of epidural EEG and subcutaneous EMG signals recorded from a freely behaving animal; the record shows a sleep-wake transition in the middle of the clip, demonstrating our method’s ability to record multi-channel EEG, EMG and monitor behavior simultaneously. Different EEG channels are colored in consistency with all other figures and the EMG channel is in black. Each EEG channel is identified with a two-letter label indicating its position: F – frontal, P – parietal, O – occipital, L – left, and R – right. (**b**) A top-view of the mouse brain showing positioning of the epidural electrodes, same as in Fig. 1c. (**c**) Epoched auditory ERPs recorded in channels FR (orange), PR (green) and OR (purple) from a wild type animal. Thin, transparent curves show individual trials (raw data) and thick, solid curves the average over all trials; the vertical gray bar marks the duration of the stimulus. (**d**) The mean spatio-temporal auditory ERP, same as in (**c**), over the cortical surface, visualized as a stack of spatially interpolated color maps through time. The gray box marks the duration of the stimulus. (**e**) Linear projections of ERPs onto the plane determined by the 3 grand averages (i.e. population means, constituting 2 spanning waveforms, #1 and #2) of channel FR, PR and OR, as shown in (**c**), color code consistent with all other figures. Each single trial of ERP is represented by a small, light-colored dot, and their grand averages by the white circles; colored ellipses mark the 1 σ boundaries of the covariances across all trials. Illustrated ERP trials are pooled from all 18 mice used in this study, which contained 9 wild type and 9 *Sp4* hypomorphic animals. (**f**) The same projection plot as in (**e**), except that the single-trial data are replaced by the 1 σ boundaries of covariances (colored ellipses) for each of the 18 animals.

We first asked whether the introduction of multiple epidural cortical recording sites provided significant spatial information in ERP characterization. The time-domain signals across different channels (Fig. 2a) were noticeably modulated. Furthermore, the grand averages of epoched ERPs had distinct waveforms along the neuraxis (Fig. 2b,c,e) and were largely symmetric bilaterally (Fig. 2d). This was observed in mice with different genetic backgrounds; a representative spatio-temporal ERP is visualized in Fig. 2d. Thus, we were able to obtain a full spatio-temporal description of sensory ERPs at a high signal-to-noise-ratio (Fig. 2c,d).

Next, we asked whether distinctive spatio-temporal waveforms could be reproducibly obtained across different animal subjects. Single-trial ERPs from all channels in 18 animals (9 wild type and 9 *Sp4* hypomorphic mice) were analyzed and the waveforms of different channels formed distinct clusters with significant separations (1σ between frontal and parietal, and 2σ between occipital and the others, Fig. 2e). The waveforms of the same channel across different animals were more similar than those of different channels in the same animal (Fig. 2f). The distinct spatio-temporal structure observed in sensory ERPs was common to many animals (even to those of different genotypes), providing additional confirmation that our technique had sufficient accuracy to reliably characterize the spatio-temporal structure of sensory ERPs.

**Figure 3:**
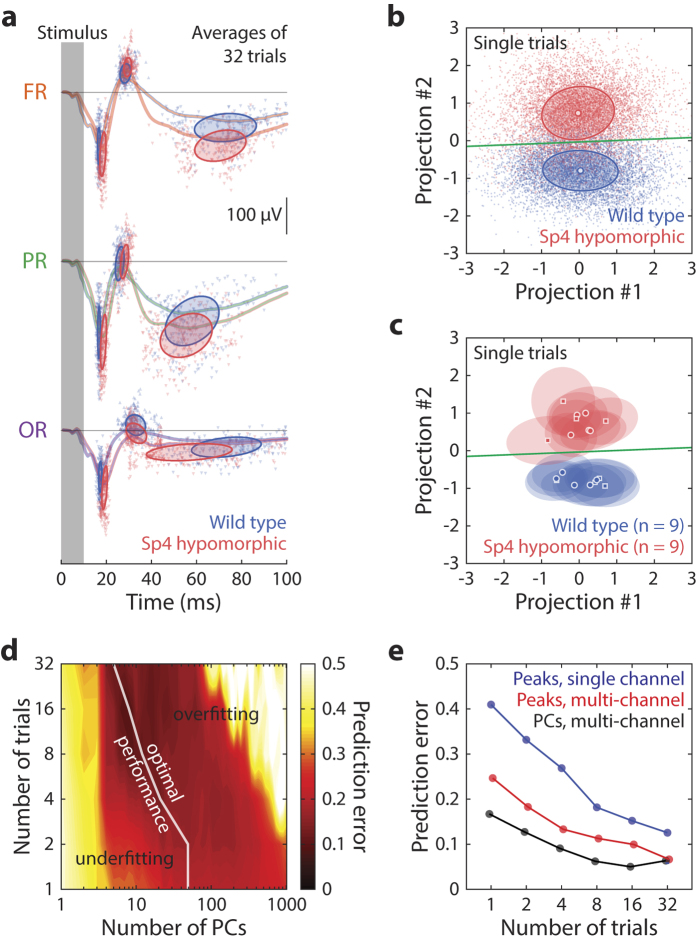
Characterizing time-domain ERP signatures unique to the mouse model under test. (**a**) Illustration of ERP peak feature extraction used in traditional methods. Grand average ERPs from channels FR, PR and OR are plotted as colored curves, the grand average of wild type animals in blue and that of *Sp4* hypomorphic mice in red. ERP peaks N1, P1, N2 detected from averages of 32-trial blocks are shown by triangular symbols, with upward-pointing triangles representing positive peaks and downward-pointing ones negative peaks. Ellipses mark the 1 σ boundaries of the covariances of the latency and magnitude of the detected peaks. (**b**) Linear projections of single-trial, full spatio-temporal ERPs (small, transparent dots) on to a plane, spanned by 2 waveforms (#1 and #2) containing the Fisher discriminant trained from the first 50 principal components. Illustrated data were pooled from all 18 mice used in this study, which contained 9 wild type and 9 *Sp4* hypomorphic animals. Circular symbols and ellipses represent the grand averages and 1 boundaries of the covariances for each genotype groups. Green line marks the decision boundary for classification of the genotype with the least prediction error. (**c**) The exact same projection plot as (**b**), except that individual trials are replaced by the mean (circles and squares, representing female and male animals respectively) and covariances (ellipses as 1 σ contours) of individual animals. (**d**) Cross-validation was performed to determine the optimal number of features (PCs) to use for classification of genotype. For each number of trials to average (vertical axis), the optimum number of features (white line) giving the least prediction error was used for building the linear classifier. (**e**) Performance of genotype classification based on ERP features is plotted against the number of trials to average. Three methods are illustrated: peaks of a single channel (blue), peaks of multiple channels (red) and PCs of multiple channels (black). Data from 5 channels, FL, FR, PR, OL and OR were used for the multi-channel cases, and channel FR was used for the single channel case.

The next question was whether the spatio-temporal information could be used to identify features of ERPs unique to the mouse model and, if so, whether the new method had any advantages over existing approaches. We first extracted time-domain features of the ERPs and then used them to train classifiers that could predict an animal’s genotype (in this case wild type versus *Sp4* hypomorphic) based on observed ERP. We further quantified whether the prediction based on multi-channel features was better than that from single-channel features. Two types of features were extracted: traditional peak components (Fig. 3a) and principal components (PCs) of waveforms. Classifiers based on linear discriminant analysis (LDA) were trained and cross-validated. Both conventional peak components and PCs had distinct structures related to the animal’s genotype (Fig. 3a–c). However, waveform PCs achieved a higher degree of resolving power for single-trial ERPs (Fig. 3b,c), which in the case of peak components could only be achieved by trial-averaged ERPs (Fig. 3a). Using peak features from multiple channels significantly improved prediction, which was further improved by using multi-channel PCs (Fig. 3e), based on quantifying prediction error in validation tests. The features combined all EEG channels in our analysis, though Figs 2 and 3 only illustrated ERPs from the right hemisphere for the sake of clarity. Our analysis revealed that the animal’s genotype could be predicted with less than 10% error rate (50% was pure chance) by multi-channel waveforms of only 4-trial averages, and the error was about 28% using conventional single-channel peak detection (Fig. 3e). The gain of resolving power by using full waveforms versus detected peaks as features suggested a significant amount of information discriminant of *Sp4* hypomorphic brain states was contained in ERP structures other than its peak components. Thus, our new technique provided additional information about the diseased brain state compared with other ERP approaches.

Finally, by projecting out the temporal component using Fisher discriminant analysis, our method allowed generating maps of the cortical surface that demonstrated genotype-specific spatial ERP signatures. The spatial patterns discriminant of the *Sp4* hypomorphic genotype showed altered polarity and amplitude, which likely reveals distinct biological mechanisms at the neural circuit level.

## Discussion

The ERP is an assay of neural circuit physiology and cognitive function. Reverse-translation has been hindered by the lack of scalable methods for accurate, robust and convenient ERP measurements in mice. We have demonstrated a new method that has high stereotaxic precision, low variability across subjects, stability over time, procedural convenience and low cost. To validate the advantages in ERP measurements for mouse models of psychiatric disorders, we characterized spatio-temporal auditory ERPs in *Sp4* hypomorphic mice^25^, a genetic model of schizophrenia that mimicked various aspects of NMDA hypofunction^25^,^26^, and identified discriminating time-domain features unique to the model. Using cross-validation, our method demonstrated high accuracy in predicting genotypes, outperforming conventional single-channel ERP component analysis in mice.

**Figure 4:**
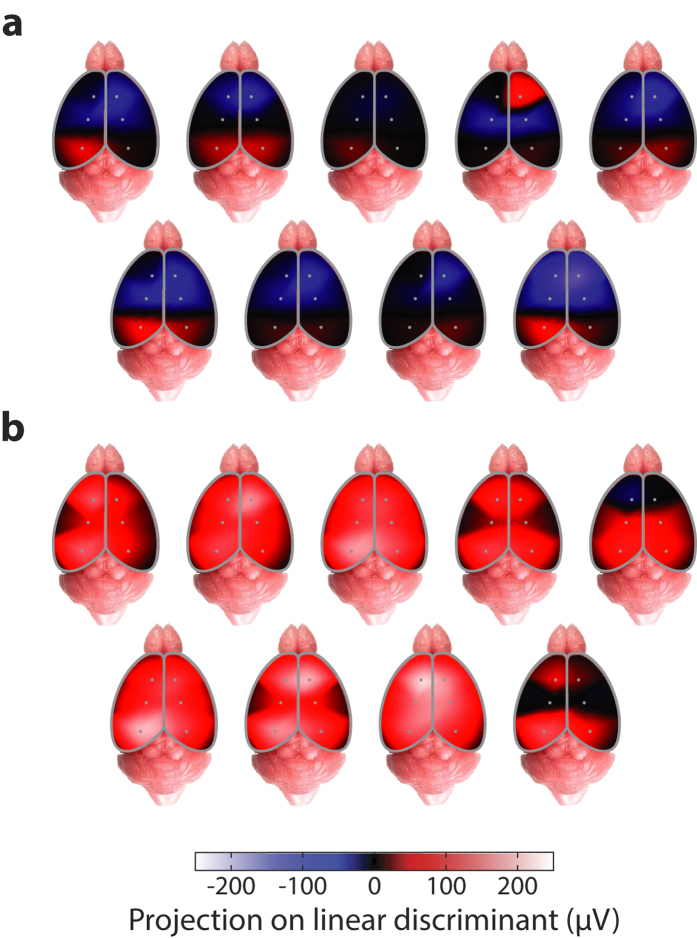
Identification of spatio-temporal ERP signatures unique to the mouse model. Interpolated cortical spatial maps of projection values onto the temporal Fisher discriminant for auditory ERPs of the *Sp4* hypomorphic model. (**a**) 9 wild type and (**b**) 9 *Sp4* hypomorphic animals are illustrated; for each group, 5 animals (upper rows) were female and 4 others (lower rows) male.

Our multi-channel epidural EEG recordings also revealed that epidural ERP waveforms over the cortical surface had distinctive spatial structures at millimeter scale (Fig. 2b–d). This suggested that, unlike the human brain where EEG signals do not significantly vary across a millimeter distance on cortex, the mouse brain had spatially structured electrical fields at a much finer scale, commensurate with its small size, as also reported previously by epicranial recordings in anesthetized mice^27^. Further, we discovered that such spatial structures in mouse epidural EEG were distinctively related to functional activities of the brain, evident from the global spatio-temporal ERP structure conserved across all animal subjects in different behavioral states (Fig. 2e,f). We also found that the quantitative description of the full spatio-temporal ERP structure had more discriminative information of the diseased brain state compared to the temporal components from a single channel (Fig. 3e). In the specific case of *Sp4* hypomorphic mice, our new methods allowed us to detect signature spatial ERP patterns, with distinct polarities and amplitudes (Fig. 4), characteristic of the disease model. This opens a new window toward the understanding of underlying biological mechanisms in future studies.

Epicranial electrode techniques have also achieved a high spatial resolution^27^,^28^,^29^ and were successfully applied to ERP measurements in anesthetized mice^27^ and seizure detection in behaving mice^30^. Intracranial techniques have been used to study epilepsy and sleep^31^,^32^,^33^. Compared to these methods, our epidural approach achieved the combination of a comparable level of accuracy, reliability and convenience in spatio-temporal ERP characterization in behaving animals, and had additional advantages for large-scale behavioral studies of psychiatric disease models.

The unique advantages of our techniques are threefold. First, we designed a minimalistic implant, in terms of material used (pre-configured electrode wires attached to a connector), weight (less than 200 mg), size (less than 8 mm protrusion) and cost (inexpensive materials and 15 minutes’ labor). Second, we developed an optimized surgical procedure (15 minutes’ labor) that matched the simplicity of the implant, and designed largely automated recording protocols compatible with behavioral experiments. These advantages allow our new method to scale up for larger behavioral studies. Last, but not least, by validating our method with the *Sp4* hypomorphic model, we found that the spatio-temporal ERP structure had sufficient resolving power to identify the genotype of an animal (i.e. wild type versus *Sp4* hypomorphic) with less than 10% error rate based on 4 trials in 10 s of ERP recording (Fig. 3e). This improvement in discriminability made it possible to rapidly characterize the temporal evolution of functional brain states, which is desirable for studying dynamical responses to pharmacological substances and makes our technique suitable for drug screening. Furthermore, by using a 3D surgical atlas^24^, it was possible to plan an implant assembly and surgery on a computer. This allowed us to foresee potential problems and avoid numerous trial-and-error iterations, enabling the rapid development of optimized procedures. More importantly, our platform supported flexible re-design of the implant and procedures.

Many laboratories have utilized wireless EEG recording techniques in experimentations with freely moving rodents. Despite significant advantages, current wireless recording devices typically have large size, heavy weight and high cost, which limit their application to mouse studies at a large scale. Our current technique aimed at striking a balance between the high channel count and sampling rate featured by wired techniques, and convenience in behavioral experimentation of wireless methods. The result was a wired solution with an implant of extremely small size, low weight, low cost and simple procedure, and at the same time having high precision and resolving power as we demonstrated here. This gave our method an advantage over existing wired or wireless methods in scaling up for large behavioral studies.

As mouse models of psychiatric disorder proliferate, better assays are needed to compare and contrast functional phenomenology at all levels of investigation. ERPs hold great promise in providing converging clues on the neuropathology of mental disorders at the neural circuit level. Thus, just as the identification and validation of ERP markers call for standardization of methodology in clinical studies^8^, powerful, scalable and reliable techniques for ERP characterization across different mouse models are similarly in need. Our new method was a first step towards meeting this challenge.

## Methods

### Implant design and preparation

Our implant contained nylon-coated stainless steel epidural EEG electrodes, which had hair-pinned exposed contacts with length of 0.5 mm from the surface of skull using 1/8 mm diameter stainless steel wires with right-angled kinks at 0.5 mm and 1.0 mm from the tip (Fig. 1d), and a nylon-coated stainless steel subcutaneous EMG electrode (all wires from PlasticOne, Roanoke, VA) channeled through an electrode interface board (EIB-8, Neuralynx, Bozeman, MT).

A 3D model of the implant (Fig. 1a,b) was designed with the assistance of the Henderson 3D surgical atlas of adult mice^24^, which contained 3D anatomical models of the whole brain and the skull. First, we mapped the desired cortical loci for epidural electrodes (Fig. 1c) to their corresponding locations on the skull (frontal: 1.0 mm anterior and 1.0 mm lateral; parietal: 1.0 mm posterior and 1.5 mm lateral; occipital: 3.5 mm posterior and 2.0 mm lateral; reference: −6.0 mm posterior and 0.0 mm lateral, all coordinates referenced to bregma). Next, we virtually assembled the implant device by connecting the loci of target craniotomies to the 3D model of the electrode interface board with electrode wires (Fig. 1a,b). Then, we placed the implant device to the most mechanically stable horizontal position (around the center of mass of the head) and adjusted the amount of vertical protrusion (less than 8 mm). Finally, we measured the resulting lengths (between 2.5 to 4.0 mm) of the electrodes for use as parameters in actual assembly.

To build the implant, we first made the electrodes separately according to their designed lengths, which were then soldered to the interface board. To facilitate the physical assembly of the implant, we created a model of the top surface of the skull from a plain piece of plastic, with desired craniotomies pre-drilled, and used it as a mold to fix the relative 3D positions of the electrodes after soldering them. The assembly was performed with optical assistance of a surgical scope or a pair of dental loupes.

### Surgical procedure

Procedures were conducted in accordance with guidelines of the National Institutes of Health and were approved by the Institutional Animal Care and Use Committee (IACUC) at the Salk Institute. In total 19 mice were used in this study, including one 3-month-old C57BL/6 mouse, 9 wild type and 9 *Sp4* hypomorphic mice (both were the F1 generation of 129S and Black Swiss genetic backgrounds) at 5–6 months of age. Animals were implanted under isoflurane anesthesia in a standard stereotaxic apparatus (Model 900, David Kopf Instruments, Tujunga, CA). A midline incision of approximately 1 cm was made; skin and muscles were retracted to expose the frontal, parietal and interparietal bones of the cranium. Stereotaxically precise craniotomies were made through a custom-made guide built from a 10 by 10 mm^2^ piece of transparent plastic with a printed crosshair marking bregma and circular holes at the target coordinates of craniotomies. The plastic guide, installed on the stereotaxic, was then manually positioned immediately on top of the skull such that the see-through crosshair coincided with the position of bregma. Next, through the guide holes, locations of the craniotomies were marked on the surface of the skull. Removing the plastic guide, craniotomies of 0.25–0.5 mm diameter were then drilled at the markings. In addition, three more craniotomies of 0.5–0.75 mm diameter were drilled for matching jewelry screws; the positions of these three screws did not need to be precise because they simply provided mechanical support (we found the arrangement of one screw on the frontal bone immediately lateral to metopic suture and two others on the interparietal cranium approximately 3 mm lateral provided adequate mechanical stability). Then, the screws were so installed that they penetrated to about 0.5 mm from the surface of the skull. Next, the implant, sterilized with 70% alcohol, was fitted on the skull with the epidural electrodes penetrating through designated craniotomies at 0.5 mm depth (Fig. 1d), exactly as it was on the model skull when it was assembled; the subcutaneous EMG electrode was placed extending posteriorly under the skin and between the two interparietal screws. A small amount of superglue was applied to the surface of the skull to provide temporary mechanical fixation at this time. Dental acrylic was then dropped from all sides of the electrode wires to cover the entire space between the electrode interface board and the surface of the skull, to the extent that no wires or screws were exposed. Finally, while the dental acrylic cured, the surgical wound was treated with wide-spectrum antibiotic ointment, and the animal was subcutaneously injected with buprenorphine (0.1 mg/kg) and removed from isoflurane anesthesia to recover. The entire procedure took about 15 minutes.

After the experiments, we verified stereotaxic precision of the electrode positions by postmortem histology; the positional error by using the above techniques was within 0.5 mm, not larger than the individual variations across subjects. We did not find significant differences in brain size across genders and genotypes in the current study.

### EEG recording

EEG recording was conducted after post-operative recovery (1–2 days). We designed 1-hour ERP recording sessions during the light phase of the circadian cycle. Before each recording session, the animal was habituated to a square, transparent acrylic recording chamber 25 cm by 25 cm in size (Fig. 1e). The implant was connected to a pre-amplifier (HS-8, Neuralynx, Bozeman, MT) with an interface cable to a slip-ring commutator (SL-88-10, Dragonfly, Ridgeley, WV); EEG and EMG signals, low-cut filtered at 0.1 Hz, were amplified and digitized (sampled at 1 kHz and 16-bit precision) by the Digital Lynx system and stored on a hard drive using Cheetah software (Neuralynx, Bozeman, MT).

Auditory stimuli and corresponding synchronizing pulses were generated on the 2 stereo channels from a sound card (using Cogent Toolbox with Matlab) at 48 kHz sampling rate. The stimulus channel drove 2 field speakers mounted on the ceiling of the recording chamber and the synchronizing pulse channel digitally recorded by Digital Lynx. The stimuli used for ERPs were 10 ms square-windowed Gaussian white noise “clicks” with an intensity of 87.3 dB on top of a white noise background of 64.0 dB in intensity (sound pressure level measured near the center of a quadrant at the bottom of the recording chamber, using standard C-weighting function). Inter-stimulus interval was set at 2.5 s and typically 1000–1200 trials were administered during a recording session. Video was simultaneously captured at 30 Hz frame rate through a camera mounted on the ceiling of the recording chamber in order to monitor animal behavior (Fig. 1e,f). We collected ERPs with a (−500, 500) ms time window relative to the onset of stimulus. ERP epochs with extreme values (absolute value greater than 1 mV) were rejected.

### ERP characterization and validation

Waveforms within the (0, 250) ms time window were used in the analysis. Single-trial ERPs and averages of 2, 4, 8, 16 and 32 trials were used for feature extraction. Conventional ERP features, i.e. amplitudes and latencies of the first 3 peak components were detected for each channel. In addition, principal component analysis was applied to ERPs of all channels, and principal components (PCs), ranking by variance from high to low, were used as waveform features.

Linear discriminant analysis (LDA) was used to identify the spatio-temporal signatures in the extracted features differentiating *Sp4* hypomorphic and wild type animals. Each sample of the LDA was a concatenation of waveforms across all EEG channels in response to a single trial of stimulus presentation. For training and validating the classifier, all samples from all animals were used, and were separated into two genotype groups. Linear classifiers based on the projection values onto the Fisher discriminants that minimized prediction error were determined for inference of genotype based on ERP features.

Leave-one-out cross validation was performed: the genotype of each animal was predicted with the classifier trained on data from the remaining animals in the dataset; this way the performance of the classifier could be objectively assessed by making predictions on data it had never encountered in training. In order to prevent overfitting, the optimal number of PCs were determined by minimizing prediction error in cross-validation; for example, optimal classification was achieved on single-trial ERPs by using the first 50 PCs and on 32-trial averages by using the first 5 PCs (Fig. 3d). The aggregate error was used to quantify performance of the prediction of genotype based on ERP features (Fig. 3e).

All analysis was performed with Matlab (Mathworks, Natick, MA).

## Additional Information

**How to cite this article**: Wang, X. *et al.* Characterization of spatio-temporal epidural event-related potentials for mouse models of psychiatric disorders. *Sci. Rep.* **5**, 14964; doi: 10.1038/srep14964 (2015).
